# Simian immunodeficiency virus and storage buffer: Field-friendly preservation methods for RNA viral detection in primate feces

**DOI:** 10.1128/msphere.00484-23

**Published:** 2023-11-30

**Authors:** Tessa H. C. Wilde, Rajni Kant Shukla, Christopher Madden, Yael Vodovotz, Amit Sharma, W. Scott McGraw, Vanessa L. Hale

**Affiliations:** 1 Department of Anthropology, The Ohio State University, Columbus, Ohio, USA; 2 Department of Veterinary Biosciences, The Ohio State University, Columbus, Ohio, USA; 3 Department of Veterinary Preventive Medicine, The Ohio State University, Columbus, Ohio, USA; 4 Department of Food Science and Technology, The Ohio State University, Columbus, Ohio, USA; University of Georgia, Athens, Georgia, USA

**Keywords:** simian immunodeficiency virus, RNA virus, preservation methods, *Cercocebus atys*, non-invasive, non-human primate, feces, storage buffer

## Abstract

Wild non-human primates carry many types of RNA viruses, including simian immunodeficiency virus (SIV), simian foamy virus, simian T-cell leukemia virus, and hepatitis C virus. These viruses can also infect humans via zoonotic transmission through handling and consumption of primate bushmeat. Characterizing viral prevalence and shedding in natural hosts is critical to understand infection and transmission risks within and between primate species. Here, we sought to identify a robust “field-friendly” method (i.e., without freezing or refrigeration) for preserving viral RNA, specifically SIV, in primate fecal samples. Fecal samples were collected from a mantled guereza colobus (*Colobus guereza*) housed at the Columbus Zoo and Aquarium. Samples were homogenized and inoculated with three concentrations (low, medium, and high) of inactivated SIV and preserved in four different storage buffers (DNA/RNA Shield, RNA*later*, 95% Ethanol, and Viral Transport Medium). SIV viral RNA was then extracted from samples at four time points (1 week, 4 weeks, 8 weeks, and 12 weeks) to determine the efficacy of each buffer for preserving SIV RNA. Quantitative reverse transcriptase polymerase chain reaction (RT-PCR) was used for the detection and quantification of viral RNA. At all concentrations, DNA/RNA Shield yielded the highest average SIV virion concentrations. We then successfully validated this approach using fecal samples from known SIV-positive and SIV-negative sooty mangabeys (*Cercocebus atys*) housed at Emory National Primate Research Center. Our results indicate that DNA/RNA Shield is an optimal “field-friendly” buffer for preserving SIV RNA in fecal samples over time and may also be effective for preserving other RNA viruses in feces.

**IMPORTANCE:**

Simian immunodeficiency virus (SIV), which originated in African monkeys, crossed the species barrier into humans and ultimately gave rise to HIV and the global HIV/AIDS epidemic. While SIV infects over 40 primate species in sub-Saharan Africa, testing for RNA viruses in wild primate populations can be challenging. Optimizing field-friendly methods for assessing viral presence/abundance in non-invasively collected biological samples facilitates the study of viruses, including potentially zoonotic viruses, in wild primate populations. This study compares SIV RNA preservation and recovery from non-human primate feces stored in four different buffers. Our results will inform future fieldwork and facilitate improved approaches to characterizing prevalence, shedding, and transmission of RNA viruses like SIV in natural hosts including wild-living non-human primates.

## INTRODUCTION

Wild non-human primates can carry many types of RNA viruses, including simian immunodeficiency virus (SIV), simian foamy virus, simian T-cell leukemia virus, and hepatitis C virus. These viruses can also infect humans via zoonotic transmission through handling and consumption of primate bushmeat. Characterizing viral prevalence and shedding in natural hosts is critical to understand infection and transmission risks within and between primate species. HIV was introduced into human populations through zoonotic transmission of the SIV from African primates, leading to a global epidemic and ongoing worldwide public health issue (1). SIV occurs naturally in over 40 primate species in sub-Saharan Africa, and these viruses have crossed species barriers on multiple occasions, leading to the spread of HIV-1 and HIV-2 in humans populations (2). Humans continue to be exposed to these RNA viruses through handling and consumption of primate bushmeat, so it is critical to investigate wild-living non-human primate populations to understand SIV prevalence, shedding, and transmission in natural hosts (3, 4). Importantly, the COVID-19 pandemic also made us globally aware of the value and need for RNA virus surveillance in wild animal populations to assess health risks to humans and animals (5–8).

Assessing the presence and abundance of RNA viruses in wild primate populations can be challenging. Biological samples must be collected non-invasively in many cases, and many field stations do not have access to a freezer or other laboratory equipment necessary to properly store and analyze these samples. Ling and colleagues (9) were one of the first groups to investigate the sensitivity of SIV detection in primate fecal samples. They tested fecal samples from laboratory-housed sooty mangabeys (*Cercocebus atys*) for SIV viral RNA and determined that real-time reverse transcriptase polymerase chain reaction (RT-PCR) detected the presence of SIV viral RNA in fecal samples from about 50% of the positive mangabeys. They went on to test this method in the field and were able to confirm one case of SIV, out of 61 samples, in a population of wild-living sooty mangabeys in Sierra Leone (10). Their samples were frozen in the field, which is not feasible in every field situation.

Additional studies have focused on red colobus monkeys (*Piliocolobus badius*) and sooty mangabeys (*Cercocebus atys*) in Taï National Park, the likely site of origin for the HIV-2 epidemic (11, 12). Based on fecal testing, these studies estimated a 50% to 60% prevalence of SIV in these wild populations.Importantly, these estimates assumed a 50% true positive detection rate in feces based on the prior studies in laboratory-housed sooty mangabeys (9). Additionally, both studies tested only for the presence or absence of the SIV virus and utilized *RNAlater* (Thermo Fisher), a storage buffer that allows fecal samples to be stored at ambient temperature for a short period of time. *RNAlater* is increasingly used to store fecal samples in the field; however, according to *RNAlater*’s guidelines, RNA quality may begin to degrade after just 1 week of storage, if not frozen. Many field studies involve weeks or months in the field, so testing storage methods with a range of ambient stability time is useful for researchers lacking freezer access in the field.

**Fig 1:**
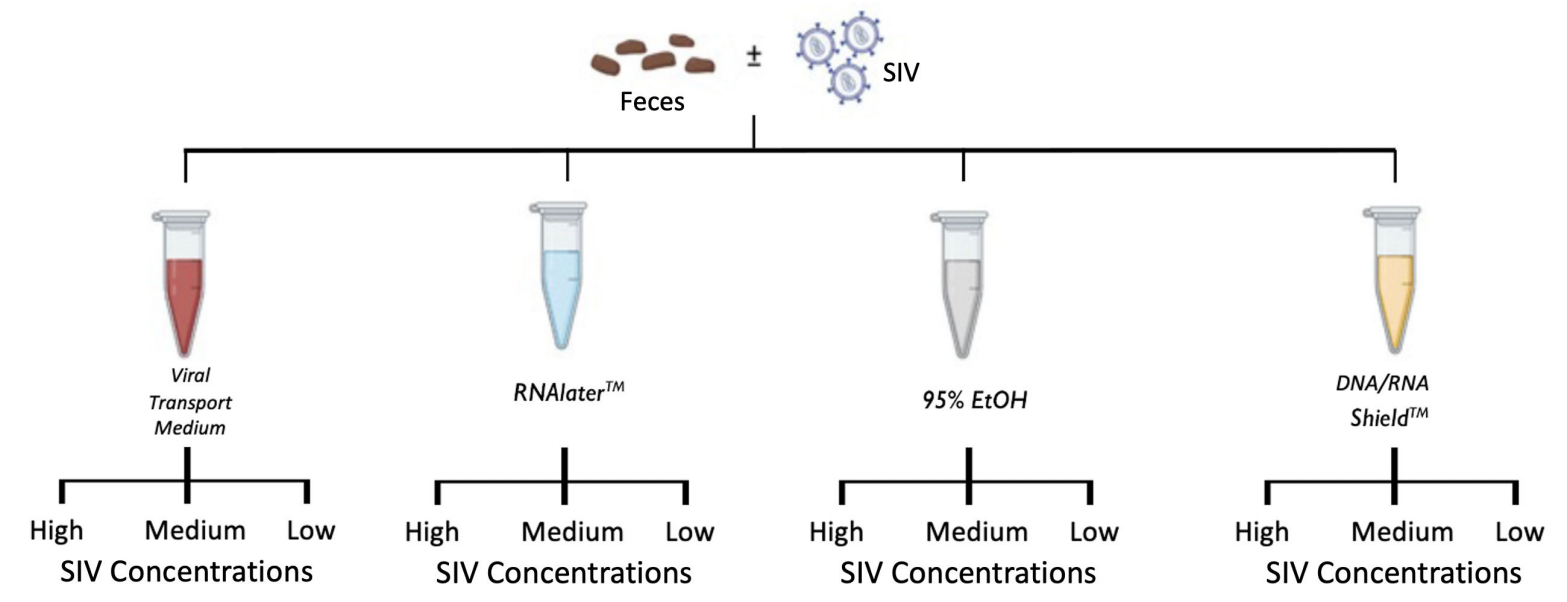
Experimental design. A single homogenized fecal sample from a colobus monkey was aliquoted into four different storage buffers (Viral Transport Medium, RNA*later*, 95% Ethanol, and DNA/RNA Shield) and inoculated with high (300,000 virions/mL), medium (30,000 virions/mL), or low (3,000 virions/mL) concentrations of SIV. RNA was then extracted from each sample at Weeks 1, 4, 8, and 12.

Here, we tested multiple “field-friendly” methods (i.e., those not requiring freezing or refrigeration) for preserving viral RNA, specifically SIV, in primate fecal samples. The storage buffers we tested included the following: DNA/RNA Shield, RNA*later*, 95% Ethanol, and Viral Transport Media (VTM). Fecal samples in each buffer were inoculated with three concentrations of SIV virus: 300,000 virions/mL (high), 30,000 virions/mL (medium), and 3,000 virions/mL (low). These concentrations fall within the range of SIV concentrations detected in the plasma of naturally infected SIV-positive primates [<500 copies/mL to greater than 2 × 10^6^ copies/mL (9)]. SIV viral RNA was then extracted from samples at four time points (1 week, 4 weeks, 8 weeks, and 12 weeks) (Fig. 1). We aimed to determine which buffer was the most effective at preserving viral RNA at different time intervals. Additionally, we (i) characterized the threshold of detection and (ii) quantified the amount of SIV viral RNA present in our study samples.

## RESULTS

### SIV virion concentrations by buffer

**Fig 2:**
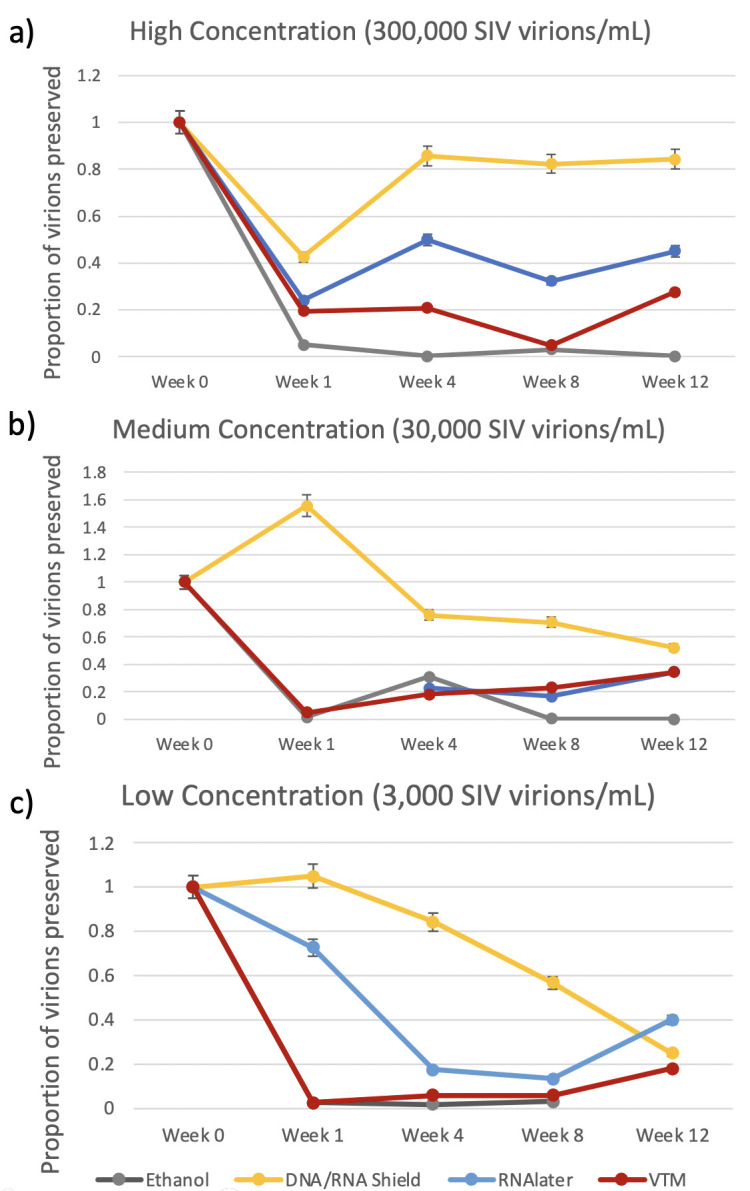
SIV yield by buffer and time. Percentage yield of SIV virions (compared with spiked in viral load) extracted from fecal samples and quantified via RT-PCR. Fecal samples were spiked with (**a**) high (300,000 virions/mL), (**b**) medium (30,000 virions/mL), and (**c**) low (3,000 virions/mL) concentrations of SIV and preserved in four storage buffers (95% Ethanol, DNA/RNA Shield, RNA*later*, and Viral Transport Medium) for up to 12 weeks.

SIV virion concentrations varied significantly by storage buffer but not significantly over time (Kruskal-Wallis: buffer *P* = 0.025; time *P* = 0.322). These results were driven by the highest and lowest performing buffers which were DNA/RNA Shield and ethanol, respectively [Dunn’s test: DNA/RNA Shield vs. Ethanol: *P* = 0.004; Ethanol vs. RNA*later: P* = 0.022; all other pairwise comparisons between buffers were not significant (Table 1)]. At all SIV concentrations (high, medium, and low), DNA/RNA Shield preserved the greatest percentage of SIV virions, followed by RNA*later*, viral transport media, and ethanol, respectively (Fig. 2). For example, at the high SIV concentration, DNA/RNA Shield preserved 84% of the SIV virions at 12 weeks, while RNA*later* preserved only 45% of the SIV virions at 12 weeks.

**TABLE 1:** *P*-values from Dunn’s pairwise test comparing SIV virion percentage yields by storage buffer^*a*^

	RNA*later*	VTM	95% Ethanol
DNA/RNA Shield	0.556	0.148	<0.004^*b*^
RNA*later*	NA	0.409	0.022^*b*^
VTM	NA	NA	0.124

^*a*^ All concentrations and time points were combined in this analysis. Percent yields were based on the original amount of SIV spiked into each sample.

^*b*^ Significant *P*-value.

### SIV virion concentrations over time

We did not observe any significant differences in virion concentrations over time (Weeks 1–12) when we compared samples within and between buffers. However, our sample size was small, and we had limited power to detect potential differences. In a few Week 1 samples, we unexpectedly observed lower SIV virion concentrations (non-significant) as compared with subsequent time points (Weeks 4, 8, or 12).

### Validation cohort

To validate our approach, we then collected blood and fecal samples from SIV-positive (*n* = 5) and SIV-negative (*n* = 5) sooty mangabeys housed at Emory National Primate Research Center (NPRC). Blood testing confirmed the SIV status of these 10 mangabeys. No SIV viral RNA was detected in the fecal samples of the SIV-negative mangabeys. In the SIV-positive mangabeys, we detected SIV viral RNA in two of the five monkeys (Table 2). The SIV viral load detected in both positive fecal samples was low (<1,000 copies/mg), and both fecal samples were associated with high serum viral loads (>120,000 copies/mL). SIV-positive mangabeys in which no SIV viral RNA detected in feces all had low serum viral loads (<32,000 copies/mL).

**TABLE 2:** Average cycle threshold (Ct) value and estimated SIV viral load for fecal and blood samples collected from SIV+ and SIV− sooty mangabeys (*Cercocebus atys*) at Emory National Primate Research Center.

Individual	SIV status	Average Ct value	Estimated fecal viral load (copies/g)	Estimated serum viral load (copies/mL)
FEb1	+	34.36	898	222,512
FGw	+	35.59	377	120,558
FDv	+	>40	0	9,399
FBy	+	>40	0	31,922
FOt	+	>40	0	30,086
FAa1	−	>40	0	0
FBw	−	>40	0	0
FGa1	−	>40	0	0
FHy	−	>40	0	0
FHz	−	>40	0	0

## DISCUSSION

African non-human primates can carry many types of RNA viruses and are natural hosts for over 40 strains of SIV. Cross-species transmission of SIV from non-human primates to humans lead to the HIV/AIDS pandemic (2). HIV and SIV viral loads are also the best predictor of AIDS progression in humans and macaques. As such, it is vital to characterize both the presence of SIV and the dynamics of SIV viral load in wild primate populations to better understand infection and transmission risks (13, 14). However, preservation and detection of RNA viruses in non-invasively collected primate fecal samples can be challenging. In this study, we tested multiple “field-friendly” storage buffers and determined that DNA/RNA Shield was most effective in preserving SIV viral RNA in fecal samples. We then validated this approach in SIV-positive and negative sooty mangabeys from Emory National Primate Research Center (NPRC).

SIV viral RNA was observed at the greatest abundances in all samples stored in DNA/RNA Shield at all times points and SIV concentrations as compared with all other buffers (with a single exception: RNA*later*, Week 12, low SIV concentration) (Fig. 2c). In two DNA/RNA Shield samples, we observed a viral RNA yield above expected (>100%) based on the amount that was spiked into each sample: Week 1, medium SIV concentration, 155% yield; Week 1, SIV low concentration, 105% yield. This may have been due to a non-homogenous distribution of SIV virions in these two sample aliquots.

RNA*later* has become one of the most widely used buffers for storing non-human primate fecal samples collected in the field; however, our results indicate that DNA/RNA Shield is a more effective choice for preserving viral RNA in fecal samples (Table 3). DNA/RNA Shield manufacturer guidelines indicate that samples can be stored at room temperature for up to 4 weeks but should then be frozen at −80°C to prevent further sample degradation. At week 4, DNA/RNA Shield preserved between 76% and 86% of the SIV virions spiked into each sample at all concentrations. Notably, we observed a non-significant decrease in virion concentrations over time in DNA/RNA Shield at medium and low SIV concentrations. However, these samples were kept at room temperature for the duration of the study (12 weeks), which was well past the manufacturer’s recommended shelf stable timeframe (1 month). Had we followed manufacturer protocols, it is possible that virion concentrations would have stabilized to an even greater degree, rather than continuing to decline over time.

**TABLE 3:** Comparison of buffers for preservation of RNA viruses in fecal samples

Buffer	Manufacturer	Cost per mL	Fecal sample-to-buffer ratio (mg:mL)	Time shelf stable (at 25°C)	Percent yield (high concentration, Week 4)	Considerations	Examples of use in previous studies
DNA/RNA Shield	Zymo	~$1.90	100:1	>30 days	85.7%	Incompatible with some non-Zymo kits and workflows.	(15)
RNA*later*	Thermo Fisher	~$1.10	200:1	1 week	50.0%	Usable with most commercially available kits and workflows.	(16, 17)
Viral Transport Medium	^*a*^	~$0.30	1,000:1	Not shelf stable	20.9%	Samples must be frozen.	(15, 18)
95% Ethanol	^*a*^	<$0.10	1,000:1	Indefinitely	0.1%	Shelf stable for DNA, but not shelf stable for RNA.	(19)

^*a*^ Available from multiple manufacturers.

We also observed that a few Week 1 samples had moderately (but non-significantly) less SIV viral RNA as compared with subsequent time points (Weeks 4, 8, or 12). While the SIV virions should have experienced the least degradation at Week 1 and SIV viral RNA yield should have been the highest at this time point, it is possible that the extracted viral RNA in these samples degraded over time in the freezer. Week 1 viral RNA was stored frozen longer than samples from all other time points. These non-significant differences could also be attributed to minor variation in sample aliquoting and measurement.

Interestingly, both DNA/RNA Shield and RNA*later* produced higher percentage yields of SIV viral RNA as compared with VTM, which was used as our “gold standard” (Fig. 2). The Centers for Disease Control and Prevention (CDC) recommends VTM for the storage of RNA viruses, such as SARS-CoV-2, for “efficient diagnosis” (20). However, similar to our results, previous studies comparing DNA/RNA Shield to VTM in SARS-CoV-2 testing have observed lower viral abundances (higher Ct values) in samples stored in VTM than those stored in DNA/RNA Shield. This indicates that DNA/RNA Shield may be an even more effective alternative to the current gold standard for RNA virus preservation in biological samples (15).

We also observed consistently low viral RNA yield in samples stored in 95% Ethanol (less than 31% SIV viral RNA yield at all concentrations and time points). Ethanol is commonly used in non-human primate field studies for the long-term storage of fecal samples, and it is therefore important to understand the limitations of this buffer. However, despite its limitations, we were able to successfully detect SIV in ethanol-preserved samples at Weeks 1, 4, and 8 (Fig. 2c), indicating that RT-PCR is highly sensitive to the presence of SIV in ethanol, even at low SIV concentrations.

Based on the performance of DNA/RNA Shield in our laboratory experiment, we sought to validate these results using fecal samples from naturally infected SIV-positive and SIV-negative laboratory-housed sooty mangabeys. We did not detect SIV RNA in any of the SIV-negative mangabeys; however, we successfully detected SIV and quantified viral load in fecal samples from two of the five SIV-positive sooty mangabeys. These fecal samples both had low viral loads—less than 1,000 viral copies SIV/g feces, which is considerably lower than viral loads typically found in serum (9, 21). Additionally, both positive fecal samples were associated with serum viral loads greater than 120,000 viral copies/mL. All negative fecal samples from SIV-positive mangabeys were associated with serum viral loads below 32,000 viral copies/mL. Sooty mangabey serum viral loads may range from less than 500 copies/mL to greater than 2 × 10^6^ copies/mL (9). This indicates that fecal viral shedding is lower in individuals with lower serum viral loads, making it more difficult to detect SIV in these individuals using non-invasive methods. The primary site of SIV/HIV viral replication is lymphoid tissue, where the virus enters and replicates within CD4+ T cells. These cells circulate throughout the body via the blood stream, making serum testing the most effective method for SIV/HIV testing. The gut also features an extensive array of lymphoid tissue known as gut-associated lymphoid tissue in which SIV/HIV viral replication occurs, which can result in viral shedding into the feces (22). Increased fecal viral shedding during HIV infection has been associated with gut dysfunction (23). However, as natural hosts of SIV, sooty mangabeys do not progress to AIDS [with one reported exception (24)] and do not experience gut dysbiosis or inflammation during chronic SIV infection (25). They are therefore less likely to shed large amounts of virus into the feces.

In conclusion, our results indicate that DNA/RNA Shield effectively preserved SIV viral RNA in fecal samples stored at room temperature for up to 3 months. However, given the decreases in SIV viral RNA yield that we observed (even between Weeks 1 and 4, which is within manufacturer guidelines for shelf-stable storage), samples should be shifted to a −80°C freezer as quickly as possible. These results also demonstrate that RT-PCR can be used to quantify SIV viral concentration in fecal samples, rather than simply report the presence or absence of virus, which may help to improve our knowledge of viral load and viral shedding in wild primates. Moreover, results from our validation cohort confirmed that DNA/RNA Shield preserved SIV viral RNA in fecal samples of naturally infected non-human primates, indicating the efficacy of this buffer for preserving RNA viruses in feces.

## MATERIALS AND METHODS

### Sample collection and preservation

A fresh fecal sample was collected from a single healthy SIV-negative mantled guereza colobus (*Colobus guereza*) that was housed at the Columbus Zoo and Aquarium. The sample was immediately transported on ice to The Ohio State University College of Veterinary Medicine, homogenized, and aliquoted into multiple tubes with four different storage buffers: RNA*later* (Thermo Fisher), Viral Transport Medium [Medium 199 with 0.5% fetal bovine serum(FBS)], DNA/RNA Shield (Zymo Research), and 95% Ethanol (Fig. 1). Fecal samples in each buffer were then inoculated with three concentrations of SIV virus: 300,000 virions/mL (high), 30,000 virions/mL (medium), and 3,000 virions/mL (low). These concentrations fall within the range of SIV concentrations detected in the plasma of naturally infected SIV-positive primates [<500 copies/mL to greater than 2 × 10^6^ copies/mL (9)].

The SIV virus inoculated into fecal samples originated from SIVmac239 viral stock which was generated by transfecting 2 × 106 HEK293T cells with 4 µg of SIVmac239 SpX plasmid DNA and 12 µL of Fugene 6 transfection reagent (Promega) following manufacturer’s protocol. Forty-eight hours post-transfection, virus-containing supernatant was harvested, passed through a 0.2-µm sterile filter, inactivated by treatment with 1 mM final concentration of Aldrithiol-2 (AT-2; Sigma), and concentrated ~10-fold using Amicon Ultracel 100-kDa filters (Millipore). Aliquots of SIVmac239 stocks were stored at −80°C. The viral titer of SIVmac239 stocks pre- and post-AT-2-treatment was determined by infecting TZM-bl cells and staining for β-galactosidase activity 48 hours post-infection.

Virus-inoculated fecal samples were then stored at room temperature (25°C) for up to 12 weeks, with the exception of those in VTM which were immediately stored at −80°C and considered our “gold standard” for sample preservation of RNA viruses (26). For many of these storage buffers, 12 weeks at room temperature is not recommended for optimal results, but we chose to extend our sampling to 12 weeks to mimic a long-term field study. Viral RNA was then extracted at four different time points (Weeks 1, 4, 8, and 12) using the QIAGEN RNeasy PowerMicrobiome Kit (Hilden, Germany). Negative controls were included for each buffer at each time point. Following extraction, viral RNA was frozen at −80°C.

### SIV plasmid linearization/standard curve

SIVmac239 SpX plasmid was obtained from NIH AIDS Reagent Program (Catalog No. ARP-12249). Plasmid DNA containing one copy of the SIV gene was linearized using EcoR1 (New England Biolabs, Ipswich, MA) following their restriction digest protocol (27). In brief, 2 µg of plasmid DNA was combined with 40 units of EcoR1, NEB buffer, and deionized water to a final volume of 50 µL. The resulting solution was incubated for 1 hour at 37°C. Following incubation, EcoR1 was then heat inactivated for 20 minutes at 65°C. The resulting linear DNA was then cleaned and concentrated with a commercially available kit (Zymo, Irvine, CA). The final DNA concentration was quantified via a Qubit fluorometer (Thermo Fisher, Waltham, MA) and used without further purification.

Following linearization, a standard curve was constructed via RT-PCR (Agilent, Santa Clara, CA) with copy numbers of the SIV gene ranging from 4.25 × 10^9^ copies to 85 copies using serial dilutions. RT-PCR was performed utilizing the S-GAG set of primers and probe (28)(Sigma-Aldrich, Burlington, MA) with a 1-Step Master Mix (Thermo Fisher, Waltham, MA). The cycling conditions were 48°C for 10 minutes followed by 95°C for 10 minutes and 45 cycles of 95°C for 15 seconds and 60°C for 1 minute. The standard curve generated the following equation in which *y* = Ct value and *x* = viral copies: *y* = −1.42ln(*x*) + 39.443, *R*^2^ = 0.9978 (Fig. S1**)**.

### Quantification of SIV viral load via RT-PCR

SIV viral RNA was amplified via RT-PCR using GAG primers and probe. Samples were run in triplicate on a 7500 Fast PCR Instrument. RT-PCR cycling conditions were as follows: 10 minutes at 48°C, 10 minutes at 95°C, 15 seconds at 95°C, and 1 minute at 60°C, for 45 cycles. Negative controls for each buffer at each time point and for the RT-PCR amplification process were included in the RT-PCR run. Triplicate Ct values for each sample were then averaged to estimate the SIV viral load. Replicates with Ct values that exhibited greater than 3% variation were excluded from analysis, and all samples required at least two replicates within 3% variation to be included in analysis. The lowest Ct value associated with any negative control was 36.4; therefore, any true (non-control) sample that had a Ct value above 36.4 was excluded from analysis (Table S1). SIV virion concentrations and percent yield expected were then calculated based on Ct values and the standard curve described above. We then compared SIV virion concentrations between buffers and over time using Kruskal-Wallis one way analysis of variance followed by pairwise Dunn’s tests.

### Validation cohort

We collected blood and fecal samples from five SIV-positive and five SIV-negative sooty mangabeys (*Cercocebus atys*) housed at Emory NPRC in Atlanta, GA. Fresh fecal samples were collected opportunistically within 1 hour of defecation after individuals were isolated in cages for their annual veterinary exams. All fecal samples were placed in DNA/RNA Shield and stored at −80°C until RNA extraction. Concurrent blood samples were collected from the mangabeys during their annual exams to confirm SIV status. Viral RNA was extracted from fecal and blood samples using the Zymo Quick-RNA Viral Kit. RT-PCR was run on all samples using the methods described above, and Ct values were used to quantify the SIV viral load.

## SUPPLEMENTAL MATERIAL

Supplemental Material msphere.00484-23-s0001.docx Figure S1 and Table S1.

ASM does not own the copyrights to Supplemental Material that may be linked to, or accessed through, an article. The authors have granted ASM a non-exclusive, world-wide license to publish the Supplemental Material files. Please contact the corresponding author directly for reuse.

## References

[1] Hahn BH, Shaw GM, De Cock KM, Sharp PM. 2000. AIDS as a zoonosis: scientific and public health implications. Science 287:607–614. doi:10.1126/science.287.5453.607 10649986

[2] Apetrei C, Robertson DL, Marx PA. 2004. The history of SIVS and AIDS: epidemiology, phylogeny and biology of isolates from naturally SIV infected non-human primates (NHP) in Africa. Front Biosci 9:225–254. doi:10.2741/1154 14766362

[3] Ahuka-Mundeke S, Ayouba A, Mbala-Kingebeni P, Foncelle C, Mubonga M, Ndimbo-Kumugo S-P, Lunguya-Metila O, Mbenzo-Abokome V, Muyembe-Tamfum J-J, Delaporte E, Peeters M. 2017. High prevalences and a wide genetic diversity of simian retroviruses in non-human primate bushmeat in rural areas of the democratic republic of Congo. Ecohealth 14:115–115. doi:10.1007/s10393-017-1223-3 28258524

[4] Aghokeng AF, Ayouba A, Mpoudi-Ngole E, Loul S, Liegeois F, Delaporte E, Peeters M. 2010. Extensive survey on the prevalence and genetic diversity of SIVs in primate bushmeat provides insights into risks for potential new cross-species transmissions. Infect Genet Evol 10:386–396. doi:10.1016/j.meegid.2009.04.014 19393772 PMC2844463

[5] Hale VL, Dennis PM, McBride DS, Nolting JM, Madden C, Huey D, Ehrlich M, Grieser J, Winston J, Lombardi D, Gibson S, Saif L, Killian ML, Lantz K, Tell RM, Torchetti M, Robbe-Austerman S, Nelson MI, Faith SA, Bowman AS. 2022. SARS-CoV-2 infection in free-ranging white-tailed deer. Nature 602:481–486. doi:10.1038/s41586-021-04353-x 34942632 PMC8857059

[6] Ehrlich M, Madden C, Mcbride DS, Nolting JM, Huey D, Kenney S, Wang Q, Saif LJ, Vlasova A, Dennis P, Lombardi D, Gibson S, Mclaine A, Lauterbach S, Yaxley P, Winston JA, Diaz-campos D, Pesapane R, Flint M, Flint J, Junge R, Faith SA, Bowman AS, Hale VL. 2023. Lack of SARS-CoV-2 viral RNA detection among a convenience sampling of Ohio wildlife. Companion, and Agricultural Animals. doi:10.3390/ani13162554 PMC10451347 37627345

[7] Delahay RJ, de la Fuente J, Smith GC, Sharun K, Snary EL, Flores Girón L, Nziza J, Fooks AR, Brookes SM, Lean FZX, Breed AC, Gortazar C. 2021. Assessing the risks of SARS-CoV-2 in wildlife. One Health Outlook 3:7. doi:10.1186/s42522-021-00039-6 33834160 PMC8024038

[8] Gwenzi W, Skirmuntt EC, Musvuugwa T, Teta C, Halabowski D, Rzymski P. 2022. Grappling with (re)-emerging infectious zoonoses: risk assessment, mitigation framework, and future directions. International Journal of Disaster Risk Reduction 82:103350. doi:10.1016/j.ijdrr.2022.103350

[9] Ling B, Santiago ML, Meleth S, Gormus B, McClure HM, Apetrei C, Hahn BH, Marx PA. 2003. Noninvasive detection of new simian immunodeficiency virus lineages in captive sooty mangabeys: ability to amplify Virion RNA from fecal samples correlates with viral load in plasma. J Virol 77:2214–2226. doi:10.1128/jvi.77.3.2214-2226.2003 12525656 PMC140942

[10] Ling B, Telfer P, Reed P, Robertson DL, Marx PA. 2004. A link between SIVsm in sooty mangabeys (SM) in wild-living monkeys in Sierra Leone and SIVsm in an American-based SM colony 20:1348–1351. 10.1089/aid.2004.20.1348 15650427

[11] Locatelli S, Roeder AD, Bruford MW, Noë R, Delaporte E, Peeters M. 2011. Lack of evidence of simian immunodeficiency virus infection among nonhuman primates in taï national park, côte d'Ivoire: limitations of noninvasive methods and SIV diagnostic tools for studies of primate retroviruses. Int J Primatol 32:288–307. doi:10.1007/s10764-010-9466-7 23950618 PMC3742091

[12] Santiago ML, Range F, Keele BF, Li Y, Bailes E, Bibollet-Ruche F, Fruteau C, Noë R, Peeters M, Brookfield JFY, Shaw GM, Sharp PM, Hahn BH. 2005. Simian immunodeficiency virus infection in free-ranging sooty mangabeys (cercocebus atys atys) from the taï forest, côte d'Ivoire: implications for the origin of epidemic human immunodeficiency virus type 2. J Virol 79:12515–12527. doi:10.1128/JVI.79.19.12515-12527.2005 16160179 PMC1211554

[13] Mellors JW, Rinaldo CR, Gupta P, White RM, Todd JA, Kingsley LA. 1996. Prognosis in HIV-1 infection predicted by the quantity of virus in plasma. Science 272:1167–1170. doi:10.1126/science.272.5265.1167 8638160

[14] Staprans SI, Dailey PJ, Rosenthal A, Horton C, Grant RM, Lerche N, Feinberg MB. 1999. Simian immunodeficiency virus disease course is predicted by the extent of virus replication during primary infection. J Virol 73:4829–4839. doi:10.1128/JVI.73.6.4829-4839.1999 10233944 PMC112526

[15] Barrera-Avalos C, Luraschi R, Vallejos-Vidal E, Figueroa M, Arenillas E, Barría D, Hernández F, Mateluna C, Mena J, Rioseco C, Torrent C, Vergara C, Gutiérrez G, Quiroz J, Alarcón J, Cartagena J, Cayunao J, Mella-Torres A, Santibañez Á, Tapia S, Undurraga A, Vargas D, Wong V, Inostroza-Molina A, Valdés D, Imarai M, Acuña-Castillo C, Reyes-López FE, Sandino AM. 2022. Analysis by real-time PCR of five transport and conservation mediums of nasopharyngeal swab samples to COVID-19 diagnosis in Santiago of Chile. J Med Virol 94:1167–1174. doi:10.1002/jmv.27446 34755352 PMC8662110

[16] Gopalakrishnan RM, Aid M, Mercado NB, Davis C, Malik S, Geiger E, Varner V, Jones R, Bosinger SE, Piedra-Mora C, Martinot AJ, Barouch DH, Reeves RK, Tan CS. 2021. Increased IL-6 expression precedes reliable viral detection in the rhesus macaque brain during acute SIV infection. JCI Insight 6:e152013. doi:10.1172/jci.insight.152013 34676832 PMC8564899

[17] Wille M, Yin H, Lundkvist Å, Xu J, Muradrasoli S, Järhult JD. 2018. Rnalater is a viable storage option for avian influenza sampling in logistically challenging conditions. J Virol Methods 252:32–36. doi:10.1016/j.jviromet.2017.11.004 29129490

[18] Latorre-Margalef N, Tolf C, Grosbois V, Avril A, Bengtsson D, Wille M, Osterhaus A, Fouchier RAM, Olsen B, Waldenström J. 2014. Long-term variation in influenza A virus prevalence and subtype diversity in migratory mallards in northern Europe. Proc Biol Sci 281:20140098. doi:10.1098/rspb.2014.0098 24573857 PMC3953854

[19] Krafft AE, Russell KL, Hawksworth AW, McCall S, Irvine M, Daum LT, Connoly JL, Reid AH, Gaydos JC, Taubenberger JK. 2005. Evaluation of PCR testing of ethanol-fixed nasal SWAB specimens as an augmented surveillance strategy for influenza virus and adenovirus identification. J Clin Microbiol 43:1768–1775. doi:10.1128/JCM.43.4.1768-1775.2005 15814997 PMC1081350

[20] Prevention C for DC and. 2020. Interim guidelines for collecting, handling, and 374 testing clinical specimens from persons for Coronavirus disease 2019 (COVID-19)

[21] Palesch D, Bosinger SE, Tharp GK, Vanderford TH, Paiardini M, Chahroudi A, Johnson ZP, Kirchhoff F, Hahn BH, Norgren RB, Patel NB, Sodora DL, Dawoud RA, Stewart C-B, Seepo SM, Harris RA, Liu Y, Raveendran M, Han Y, English A, Thomas GWC, Hahn MW, Pipes L, Mason CE, Muzny DM, Gibbs RA, Sauter D, Worley K, Rogers J, Silvestri G. 2018. Sooty mangabey genome sequence provides insight into AIDS resistance in a natural SIV host. Nature 553:77–81. doi:10.1038/nature25140 29300007 PMC5843367

[22] Veazey RS, DeMaria M, Chalifoux LV, Shvetz DE, Pauley DR, Knight HL, Rosenzweig M, Johnson RP, Desrosiers RC, Lackner AA. 1998. Gastrointestinal tract as a major site of CD4+ T cell depletion and viral replication in SIV infection. Science 280:427–431. doi:10.1126/science.280.5362.427 9545219

[23] Yolken RH, Li S, Perman J, Viscidi R. 1991. Persistent diarrhea and fecal shedding of retroviral nucleic acids in children infected with human immunodeficiency virus. J Infect Dis 164:61–66. doi:10.1093/infdis/164.1.64 2056218

[24] Ling B, Apetrei C, Pandrea I, Veazey RS, Lackner AA, Gormus B, Marx PA. 2004. Classic AIDS in a sooty mangabey after an 18-year natural infection. J Virol 78:8902–8908. doi:10.1128/JVI.78.16.8902-8908.2004 15280498 PMC479084

[25] Pandrea I, Apetrei C. 2010. Where the wild things are: pathogenesis of SIV infection in African nonhuman primate hosts. Curr HIV/AIDS Rep 7:28–36. doi:10.1007/s11904-009-0034-8 20425055 PMC2824118

[26] Abdallah NMA, Zaki AM, Abdel-Salam SA. 2020. Stability of MERS-CoV RNA on spin columns of RNA extraction kit at room temperature. Diagn Microbiol Infect Dis 98:115182. doi:10.1016/j.diagmicrobio.2020.115182 32947111 PMC7441011

[27] New England Biolabs. 2023 Restriction Enzyme Digestion. NEBcloner. Available from: https://nebcloner.neb.com/#!/protocol/re/single/EcoRI

[28] Cline AN, Bess JW, Piatak M, Lifson JD. 2005. Highly sensitive SIV plasma viral load assay: practical considerations, realistic performance expectations, and application to reverse engineering of vaccines for AIDS. J Med Primatol 34:303–312. doi:10.1111/j.1600-0684.2005.00128.x 16128925

