# Supplementary material for: Simian immunodeficiency virus and storage buffer: Field-friendly preservation methods for RNA viral detection in primate feces
Source: mSphere. 2023 Nov 30;8(6):e00484-23. doi: 10.1128/msphere.00484-23 (PMC10732032; doi:10.1128/msphere.00484-23)
Supplement: Supplemental Material — Figure S1 and Table S1. [file msphere.00484-23-s0001.docx]

**Supplemental Materials**

***Simian Immunodeficiency Virus and Storage Buffer: Field-friendly preservation methods for RNA viral detection in primate feces***

TESSA H.C. WILDE, RAJNI KANT SHUKLA, CHRISTOPHER MADDEN, YAEL VODOVOTZ, AMIT SHARMA^,^ W. SCOTT MCGRAW, VANESSA L. HALE

**Figure S1:** **SIV RT-PCR standard curve.**

**Table S1: Mean cycle threshold (Ct) values and SIV virion concentrations by buffer and time.** SIV virion concentrations were determined based on Ct value (and associated viral copy number) and initial sample to buffer ratios. Percent expected yield was determined by dividing actual viral copy number by expected viral copy number, assuming that 100% of viral copies were consistently preserved over time. *Variation between Ct values exceeded 3% and these samples were excluded from analyses. **Excluded from analyses as Ct values exceeded Ct of lowest negative control at threshold of 36.4.

| Storage Buffer | Week | Concentration | Ct Value | Virion Concentration (copies/uL) | Percent expected yield |
| --- | --- | --- | --- | --- | --- |
| DNA/RNA Shield | 1 | High | 25.698 | 127,668 | 42.56% |
|  |  | Med | 27.128 | 46,623 | 155.41% |
|  |  | Low | 30.957 | 3,145 | 104.8% |
|  | 4 | High | 24.704 | 257,074 | 85.69% |
|  |  | Med | 28.149 | 22,709 | 75.70% |
|  |  | Low | 31.269 | 2,525 | 84.16% |
|  | 8 | High | 24.761 | 246,887 | 82.30% |
|  |  | Med | 28.247 | 21,199 | 70.66% |
|  |  | Low | 31.828 | 1,702 | 56.74% |
|  | 12 | High | 24.72 | 252,421 | 85.14% |
|  |  | Med | 28.675 | 15,683 | 52.28% |
|  |  | Low | 33.004 | 744 | 24.79% |
| RNA*later* | 1 | High | 25.842 | 72,057 | 24.01% |
|  |  | Med | * | * | * |
|  |  | Low | 30.812 | 2,177 | 72.55% |
|  | 4 | High | 24.802 | 149,900 | 50.00% |
|  |  | Med | 29.201 | 6,769 | 22.56% |
|  |  | Low | 32.849 | 480 | 17.28% |
|  | 8 | High | 25.427 | 96,527 | 32.18% |
|  |  | Med | 29.643 | 4,958 | 16.52% |
|  |  | Low | 33.225 | 398 | 13.26 |
|  | 12 | High | 24.950 | 135,052 | 45.02% |
|  |  | Med | 28.598 | 10,347 | 34.49% |
|  |  | Low | 31.662 | 1,197 | 39.89% |
| Viral Transport Medium | 1 | High | 26.144 | 58,270 | 19.42% |
|  |  | Med | 31.355 | 1,485 | 4.95% |
|  |  | Low | 35.669 | 71 | 2.37% |
|  | 4 | High | 26.042 | 62,614 | 20.87% |
|  |  | Med | 29.518 | 5,416 | 18.05% |
|  |  | Low | 34.384 | 176 | 5.86% |
|  | 8 | High | 28.117 | 14,522 | 4.84% |
|  |  | Med | 29.173 | 6,903 | 23.01% |
|  |  | Low | 34.420 | 171 | 5.72% |
|  | 12 | High | 25.643 | 82,901 | 27.63% |
|  |  | Med | 28.601 | 10,330 | 34.43% |
|  |  | Low | 32.802 | 536 | 17.87% |
| 95% Ethanol | 1 | High | 28.022 | 15,530 | 5.18% |
|  |  | Med | 32.831 | 525 | 1.75% |
|  |  | Low | 35.644 | 72 | 2.41% |
|  | 4 | High | 33.291 | 380 | 0.13% |
|  |  | Med | 28.756 | 9,259 | 30.86% |
|  |  | Low | 36.120 | 52 | 1.72% |
|  | 8 | High | 28.713 | 9,544 | 3.18% |
|  |  | Med | 34.469 | 166 | 0.55% |
|  |  | Low | 35.396 | 86 | 2.88% |
|  | 12 | High | 32.532 | 648 | 0.22% |
|  |  | Med | ** | ** | ** |
|  |  | Low | ** | ** | ** |
